# Genetic diversity and antimicrobial susceptibility of *Nocardia* species among patients with nocardiosis

**DOI:** 10.1038/srep17862

**Published:** 2015-12-07

**Authors:** Abodolrazagh Hashemi-Shahraki, Parvin Heidarieh, Saeed Zaker Bostanabad, Mohamad Hashemzadeh, Mohamad Mehdi Feizabadi, Dean Schraufnagel, Mehdi Mirsaeidi

**Affiliations:** 1 Department of Epidemiology, Pasture Institute of Iran, Tehran, Iran; 2 Department of Microbiology, School of Medicine, Alborz University of Medical Sciences, Alborz, Iran; 3 Biology and Microbiology Department, Islamic Azad University, Parand Branch, Tehran, Iran; 4 Health Research Institute, Infectious and Tropical Diseases Research Center, Ahvaz Jundishapur University of Medical Sciences, Ahvaz, Iran; 5 Department of Microbiology, School of Medicine, Tehran University of Medical Sciences, Tehran, Iran; 6 Division of Pulmonary and Critical Care, University of Illinois at Chicago, Chicago, IL, USA; 7 Division of Pulmonary and Critical Care, University of Miami, Miami, FL, USA

## Abstract

The aim of this multicenter study was to determine the genetic diversity and antibiotic susceptibility of clinically isolated *Nocardia* species. One hundred twenty-seven patients with nocardiosis were randomly selected from 5 provinces of Iran. Molecular diagnosis of *Nocardia* species was performed using multilocus sequence analysis of gyrase B of the β subunit of DNA topoisomerase (*gyrB*), and 16S rRNA and subunit A of SecA preproteintranslocase (*secA1*). Antimicrobial susceptibility testing was performed following the Clinical and Laboratory Standards Institute recommendations. Thirty-five *N. cyriacigeorgica*, 30 *N. asteroides*, 26 *N. farcinica*, 12 *N. otitidiscaviarum,* and 10 *N. abscessus* cultures were studied. All isolates were susceptible to linezolid. All isolates of *N. cyriacigeorgica*, *N. asteroides, N. abscessus, and N. otitidiscaviarum* were susceptible to trimethoprim-sulfamethoxazole, while 8% of *N. farcinica* isolates were resistant to this drug. All *N. otitidiscaviarum* isolates were highly resistant to imipenem, but *N. cyriacigeorgica*, *N. asteroides, N. farcinica, and N. abscessus* were only moderate resistant. The susceptibility patterns vary with different species of *Nocardia*. Resistance to trimethoprim-sulfamethoxazole in Iran is low and this drug should be first line therapy, unless drug susceptibility testing shows resistance. Linezolid also covers *Nocardia* well and could be a second line agent.

*Nocardia* can be found worldwide as a saprophytic pathogen in water, soil, decaying fecal deposits from animals and other ecological niches^1^,^2^. Only a small proportion of the currently described *Nocardia* species are known to be human pathogens that affect both immunosuppressed and immunocompetent patients^3^. Nocardial infections range from minor cutaneous lesions to severe pulmonary or central nervous system disease^1^,^2^,^3^.The incidence rates of *Nocardia* species isolation from clinical samples have been increasing worldwide in the recent decades^3^,^4^,^5^. The reason for this increase could be related to advances in culturing and improved molecular methods as well as progress in oncology, rheumatology, and transplant medicine^3^,^4^,^6^,^7^.Drug susceptibility testing of *Nocardia* isolates is recommended as a guide to therapy for cases of severe or disseminated infection, refractory cases, and those who are intolerant to treatment with sulfonamides^1^,^4^. However, there is limited information about the distribution of the different *Nocardia* species and drug susceptibility of *Nocardia* worldwide including the Middle East. The aim of this study was to determine genetic diversity and *drug* susceptibility of clinical isolates of *Nocardia* from Iran.

## Material and methods

### Organisms

**Figure 1:**
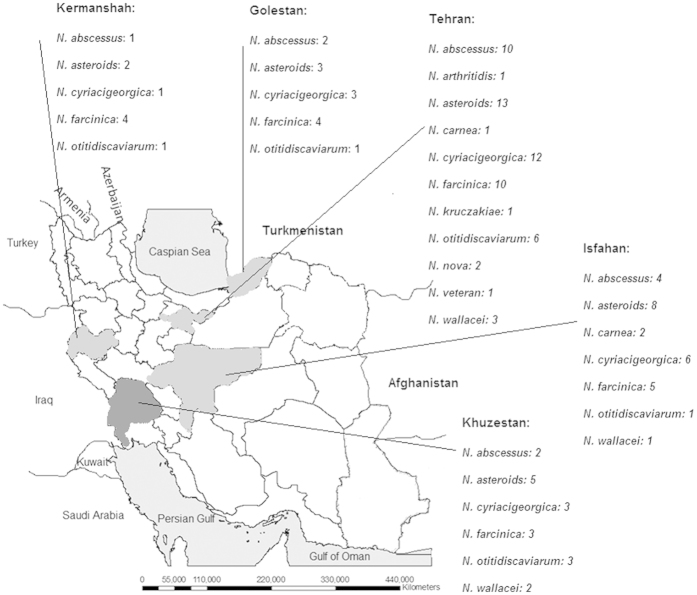
Geographic distribution of clinical isolates of *Nocardia* collected in the study. The figure was generated by AutoCAD MAP 3D“Autodesk® AutoCAD® Map 3D (http://www.autodesk.co.uk/products/autocad-map-3d/overview)” and then finalized by Photoshop CS5 software.

One hundred twenty-seven clinical isolates of *Nocardia* from different major cities of Iran were studied between 2009 and 2015; 22 were from Khosestan (southwest Iran), 47 from Tehran (central Iran), 21 from Isfahan (central Iran), 13 from Mazandaran (northwestern Iran), and 10 from Kermanshah (northeast Iran)(Fig. 1). This study approved by Ethics Committee of Ahvaz Jundishapur University of Medical Sciences (AJUMS), Ahvaz, Iran. Demographic, clinical, and microbiologic data were collected from patients’ medical records who signed the informed written consent. Isolates were sent to the Infectious and Tropical Diseases Research Center (AJUMS) for identification and subsequently antimicrobial susceptibility determination. All experimental protocols including sample collection and laboratory methods were approved by scientific committee of Health Research Institute (AJUMS).

A portion of the isolates (32specimens)were identified at the species level by multilocus sequence analysis (MLSA) of 16S rRNA, gyrase B of the ß subunit of DNA topoisomerase (*gyrB*) and subunit A of SecA preprotein translocase (*secA1*) as previously described by McTaggart and colleagues^8^ to find out the reliability of each marker for identification. The remaining isolates were identified to species level by 16S rRNA analysis because of its acceptable discriminatory power. The 16S rRNA gene was amplified using 27F primer (5′–AGAGTTTGATCCTGGCTCAG–3′) and 1525R (5′–AAGGAGGTGWTCCARCC–3′) and then was sequenced. The sequences were aligned and trimmed in BioNumerics (version 6.0.1) software (Applied Maths, Austin, TX) and were identified to species level. A representative 16S rRNA gene sequence from each of species was deposited in Genbank with KT003507-KT003513 accession numbers.

### Broth microdilution testing

The drugs amikacin, amoxicillin-clavulanate, cefepime, cefotaxime, ceftriaxone, ciprofloxacin, clarithromycin, doxycycline, gentamicin, imipenem, linezolid, minocycline, moxifloxacin, tobramycin, trimethoprim-sulfamethoxazole (TMP-SMZ), and vancomycin were selected by testing based on National Committee for Clinical Laboratory Standards (NCCLS) recommendations^9^. Microtiter plates were prepared in-house, using standard twofold dilution of all antimicrobials except ampicillin and amoxicillin-clavulanate in cation-adjusted Mueller-Hinton broth. The plates were stored at −70 °C and were thawed at room temperature immediately before use. The appropriate dilution of amoxicillin-clavulanate was freshly prepared immediately before use, then aliquoted, and placed in designated microtiter wells. Ten microliters of an inoculum with a turbidity equivalent to that of a 0.5 to 1.0 McFarland standard was dispensed into each well to give a final concentration of 10^4^ to 10^5^ CFU/mL^9^. The microtiter plates were incubated aerobically at 35 °C and were read after 3 days. Growth was examined daily by visual inspection. The minimum inhibitory concentration (MIC) was defined as the lowest concentration of the drug that inhibited visible growth. MICs at which 50% (MIC50s) and 90% (MIC90s) of isolates are inhibited were determined^9^. MIC50% and MIC90% were selected to provide an interpretation of the clinical significance of concentrations of an antimicrobial that inhibit the growth of an organism or kill it in laboratory systems (*in vitro*)^10^,^11^ and for defining the starting point for larger preclinical evaluations of novel antimicrobial agents^12^. For TMP-SMZ, the MIC was the 80% inhibition endpoint of growth compared to the control. Susceptible and resistant breakpoints were defined according to the NCCLS recommendations^9^. Quality control of the MICs was performed by the testing of NCCLS recommended reference strains, including *Enterococcus faecalis* ATCC 29212, *Nocardia abscessus* DSM 44432, *Pseudomonas aeruginosa* ATCC 27853, and *Staphylococcus aureus* ATCC 29213.The control strains were obtained from Iranian Biological Resource Center (IBRC), Tehran, Iran.

## Results

Out of 127 patients with nocardiosis 69 (54%) were females. The mean age was 47.6 (SD = 21) years. Table 1 shows the demographic information of study population. Almost half of the patients had at least one significant underlying condition, such as solid organ transplantation (11 patients, 8.7%), solid or hematologic malignancy (10 patients, 8%), HIV (13 patients, 10%), and receiving corticosteroids for rheumatologic disorders (9 patients, 7%). No known immunodeficiency was found in 56 (44%) patients. The most common symptoms were fever (52%) and cough (47%) in patients with pulmonary disease. Cavitary lesion was found on chest radiography in 18 (30%) persons with pulmonary disease. Pleural effusions occurred in 8 (14%). Lungs were the primary organ involved in 64 (50%) patients. Extrapulmonary nocardiosis included skin and soft tissue (31 persons, 24%) and central nervous system disease (brain abscess) (12 persons, 9%), and disseminated disease (18 persons, 14%). Bronchoalveolar fluid was the most common source of *Nocardia* isolation (60%). Extrapulmonary nocardiosis occurred more often in younger individuals (mean age 38.6 versus 55.8 in pulmonary group, p<0.0001) and those who were taking corticosteroids for rheumatologic disorders (8, 13% vs. 1, 2% in pulmonary group, p = 0.011). Out of 127 clinical isolates, 31 (24%) were *N. asteroides*, 25 (20%) were *N. cyriacigeorgica*, 26(21%) were *N. farcinica*,12 (9%) were *N.otitidiscaviarum*,19 (15%) were *N. abscessus*, 6(5%) were *N. wallacei,* 3 (2%) were *N. carnea,* 2 (2%) were *N.nova,* and one each were from *N. kruczkiae, N. veterana,* and *N. arthritidis*. Some of these data were published elsewhere^13^.

**Figure 2:**
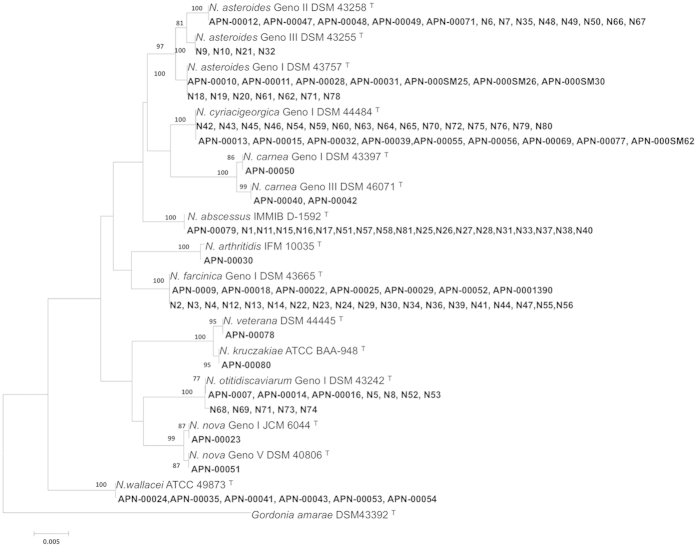
16S rRNA sequence-based phylogenetic tree of clinical isolates of *Nocardia* with those of closely related species which computed by the NJ analyses and K2P model. The support of each branch, as determined from 1000 bootstrap samples, is indicated by percentages at each node. Bar 0.005 substitutions per nucleotide position.

Figure 2 shows that all clinical isolates of *Nocardia* were clearly differentiated and formed distinct branches in phylogenetic tree based on16S rRNA. Table 2 shows that the *Nocardia* species most commonly isolated from human infections were *N. asteroides, N. farcinica* genotype I, and *N. cyriacigeorgica* genotype I. Isolates N 6, N 7, N 35, N 48, N 49, N 50, N 66 and N 67 were identified *N. asteroides* genotype II; N 9, N 10, N 21 and N 32 were grouped as *N. asteroides* genotype III, while N 18, N 19, N 20, N61, N 62, N 71, N 78 were clustered as *N. asteroides* genotype I.

### Drug susceptibility testing

Table 3 presents the MICs at which 50% (MIC50s) and 90% (MIC90s) of isolates are inhibited and the range of MICs for all *Nocardia* isolates. All *Nocardia* isolates were resistance to vancomycin.

### N. asteroides

Among the 31 isolates of *N. asteroides*, all were susceptible to TMP-SMZ and linezolid. Amoxicillin-clavulanic acid, cefepime, ceftriaxone, ciprofloxacin, imipenem, moxifloxacin, and tobramycin had moderate activity, while clarithromycin had poor activity against the clinical isolates of *N. asteroides.* The MIC90 for both linezolid and TMP-SMZ was 1 (μg/ml), but, for ceftriaxone this value was 128 (μg/ml).

### N. farcinica

All 26 isolates of *N. farcinica* were susceptible to amikacin and linezolid, and all were resistant to ceftriaxone, doxycycline, gentamicin, minocycline, and tobramycin. Two (8%) of 26 isolates were resistant to TMP-SMZ. For amikacin and linezolid the lowest concentration of MIC90 (1 μg/ml) was detected. Ceftriaxone had the highest concentration of MIC90 value between all tested antibiotics (256 μg/ml).

### N. cyriacigeorgica

The 25 *N. cyriacigeorgica* clinical isolates were susceptible to amikacin, cefepime, gentamicin, linezolid, tobramycin, and TMP-SMZ. *N. cyriacigeorgica* generally had good sensitivity to cefotaxime, ceftriaxone, clarithromycin, doxycycline, imipenem, and minocycline, but poor sensitivity to amoxicillin-clavulanic acid, ciprofloxacin, and minocycline. All isolates were resistant to moxifloxacin. The MIC90 for cefepime, linezolid, tobramycin and TMP-SMZ was 0.5 μg/ml and for amoxicillin-clavulanic acid, ceftriaxone, cefotaxime, imipenem and moxifloxacin was 64 (μg/ml).

### N. abscessus

All 19 isolates of *N. abscessus* were susceptible to ceftriaxone, gentamicin, linezolid, tobramycin, and TMP-SMZ. Cefepime, cefotaxime, doxycycline, imipenem, and minocycline showed good activity against *N. abscessus* isolates. Amikacin, amoxicillin-clavulanic acid, and clarithromycin had low activity against the clinical isolates of *N. abscessus*, and ciprofloxacin, moxifloxacin had no activity against these isolates. Ceftriaxone, gentamicin, linezolid and tobramycin had MIC90 (1 μg/ml). The MIC90 for amoxicillin-clavulanic acid and cefotaxime was 64 μg/ml and for amikacin, cefepime and imipenem was 32 μg/ml.

### N. otitidiscaviarum

All 12 isolates of *N. otitidiscaviarum* were susceptible to amikacin, linezolid, tobramycin, and TMP-SMZ, whereas there was poor activity to ceftriaxone, doxycycline, and minocycline, and all isolates were resistant to amoxicillin-clavulanic acid, imipenem. The MIC90 for amikacin, linezolid and TMP-SMZ was 0.125 μg/ml.

### N. wallacei

All 6 isolates were resistance to amikacin, clarithromycin, imipenem, moxifloxacin were susceptible to ceftriaxone, cefepime, cefotaxime, gentamicin, linezolid, tobramycin and TMP-SMZ. Amoxicillin-clavulanic acid, doxycycline and minocycline demonstrated poor activity against the isolates.

### N. carnea

All 3 isolates of *N. carnea* were resistance to amikacin, amoxicillin-clavulanic acid, ceftriaxone, ciprofloxacin, clarithromycin, imipenem, moxifloxacin were susceptible to cefepime, cefotaxime, gentamicin, linezolid, tobramycin and TMP-SMZ. Poor activity was recorded for doxycycline and minocycline.

One isolate from each species of *N. arthritidis*, *N. kruczakiae*, *N. nova* and *N. veteran* were studied for drug susceptibility tests. All of them were susceptible to amikacin, amoxicillin-clavulanic acid, ceftriaxone, ciprofloxacin, clarithromycin, imipenem, linezolid, tobramycin and TMP-SMZ.

## Discussion

*N. asteroides* was the most frequently recovered species in our study. It was followed by *N. farcinica* and *N. cyriacigeorgica*. This pattern was different between individuals with pulmonary and extrapulmonary nocardiosis, with *N. cyriacigeorgica* being the most common in extrapulmonary disease. Our study found that extrapulmonary nocardiosis occurs more commonly in younger persons (mean age 38) compared to pulmonary nocardiosis (mean age 56) and in those with rheumatologic disorders taking corticosteroids.

Although cases reports have shown *N. cyriacigeorgica*^14^, *N. asteroides* complex^15^ and *N. nova* complex^16^ in Iran, but to our knowledge, there is no report of drug susceptibility on clinical isolates of *Nocardia* from Iran as well as the Middle Eastern countries.

The lungs are the most common organ that *Nocardia* infects (up to 70%),with *N. asteroides* complex the species most often isolated from this site^17^.Yamagata and colleagues reported that patients with rheumatologic disorders who took corticosteroid were at higher risk of extrapulmonary nocardiosis^6^. Our study confirmed the higher incidence extrapulmonary nocardiosis in those taking corticosteroids before the *Nocardia* infection. This information may serve as a warning to clinicians about the risk of corticosteroids and disseminated nocardial infection.

*Nocardia* species cause a wide variety of diseases and have variable drug susceptibility profiles. Since the 1940s, the sulfonamides have been the treatment of choice for nocardiosis^1^,^18^. Later, the combination of trimethoprim with sulfamethoxazole became the most commonly recommended treatment for these infections^1^. Other therapies including amikacin, a combination of amikacin and a beta-lactam such as ceftriaxone or imipenem, and a combination with linezolid have also been suggested for therapy of patients with serious disease^1^,^19^.

Susceptibility testing of *Nocardia* isolates to the antibiotics showed that *N. cyriacigeorgica* isolates were generally sensitive to our selected antibiotics. All were susceptible to amikacin, cefepime, gentamicin, linezolid, tobramycin, and TMP-SMZ; and the majority was somewhat less susceptible to cefotaxime, ceftriaxone, doxycycline, imipenem, and minocycline. These findings are consistent with those reported by Glupczynski and colleagues^20^. Further, Larruskain and colleagues noted that *N. cyriacigeorgica* isolates from Spain were susceptible to amikacin, gentamicin, linezolid, tobramycin, and TMP-SMZ^21^.

Schlaberg and colleagues from the United States reported that *N.cyriacigeorgica* isolates were susceptible to amikacin, linezolid, tobramycin, and TMP-SMZ, and were resistant to amoxicillin-clavulanic acid, ciprofloxacin, clarithromycin, minocycline, and moxifloxacin^22^. However, our isolates were highly resistant only to amoxicillin-clavulanic acid, ciprofloxacin, and moxifloxacin. Ceftriaxone, imipenem, linezolid, and TMP-SMZ were reported as the most effective antimicrobial agents against *N.cyriacigeorgica* isolates in Taiwan^23^, which agrees with our results.

Among the 31 isolates of *N. asteroides*, linezolid and TMP-SMZ were active against all isolates while moderate susceptibility was detected for imipenem, amoxicillin-clavulanic acid, cefepime, ceftriaxone, ciprofloxacin, moxifloxacin, and tobramycin. Clarithromycin had poor activity against clinical isolates of *N. asteroides* in our study. In the preliminary evaluation of antimicrobial agents against *N. asteroides* isolates in 1984, the beta-lactams including third-generation cephalosporins were generally reported ineffective, whereas minocycline, doxycycline, and sulfamethoxazole were recommended for therapy^24^. Four years later, Wallace and colleagues showed that the most active parenteral agents against *N. asteroides* were amikacin, cefotaxime, ceftriaxone, imipenem, minocycline, and sulfonamides^25^. Although Schlaberg and colleagues found that all *N. asteroides* isolates were susceptible to amikacin, imipenem, linezolid, tobramycin, and TMP-SMZ^22^, we found less susceptibility among *N. asteroides* isolates in our study.

*N. farcinica* is more likely to have multidrug resistance and high level resistance to imipenem, ceftriaxone, clarithromycin, tobramycin, and moxifloxacin^21^,^22^. Although TMP-SMZ has been the drug of choice for the treatment of nocardiosis^7^,^18^,^24^,^25^, we found 8% (2 isolates) of *N. farcinica* were TMP-SMZ resistant. Larruskain and colleagues in Spain found 16.1%^21^, Uhde and colleagues found 42%^26^ from the United States, and Tremblay and colleagues also reported 42% TMP-SMZ resistant strains from Canada^27^. Another study from Spain, reported that 9 of 19*N. farcinica* isolates (47%) were TMP-SMZ resistant^7^. Furthermore, Lai and colleagues from Taiwan reported a low incidence (9%) similar to ours^23^, and another report from the United States also found only 2% TMP-SMZ resistance^28^ and sulfonamide and TMP-SMZ resistance was not seen in South Africa^29^. The similarity between the 2 North American countries and divergence in Europe and Iran suggests there may be geographical differences in *N. farcinica* drug sensitivity with unknown reasons. We speculate that the difference in drug susceptibility to TMP-SMZ could be related to differences in laboratory methodology and interpretation criteria. More recently, Valdezate and colleagues reported association of high-level sulfonamide resistance and the presence of plasmid-borne integrons carrying *sul* genes (*sul1* and *sul2*) in SXT-resistant *Nocardia* strains^30^.These type of integrons, and the corresponding plasmids, are commonly detected in bacteria living in different ecological niches^31^.

In our study, resistance to β-Lactams antibiotics were detected among the isolates, which might be related to a mutational change affecting the inhibitor and active site (s) in the beta-lactamase^32^.

Our *Nocardia* isolates showed moderate resistance to quinolones. Valdezate and colleagues^30^ could not find plasmid-mediated quinolone resistance genes (*qnrA*, *qnrB*, *qnrC*, and *qnr*) or the gene for the aminoglycoside acetyltransferase for modify ciprofloxacin^33^ or efflux pump *qepA*^34^ and or nucleotide changes observed in *gyrA*^35^. Further, study considering the resistance mechanisms and how antibiotic resistance spreading among *Nocardia* strains are required.

All *N. otitidiscaviarum* species were susceptible to TMP-SMZ. Our data was in agreement with those reported by others^22^,^23^. In contrast with our data, moderate resistance to TMP-SMZ (32%) among *N. otitidiscaviarum* was reported by Uhde and colleagues^26^.

*N. abscessus* were susceptible to ceftriaxone, gentamicin, linezolid, tobramycin, and TMP-SMZ in our study. The same susceptibility profile of *N. abscessus* was reported before^21^,^22^.

Linezolid, a relatively new class of antibiotics, showed extraordinary *in vitro* activity against all of the major clinically significant species of *Nocardia*^19^,^36^. Our findings are in agreement with reports from different parts of world that clearly demonstrate that linezolid is an effective alternative for the treatment of nocardiosis.

In conclusion, *N. asteroides* was the most common species isolated from pulmonary nocardiosis and *N. cyriacigeorgica* was the most frequently recovered species from extrapulmonary nocardial infections. Clinical isolates of *Nocardia* species in our study had varied drug susceptibility patterns, which were similar to what have been reported from other geographic area, with some exceptions. Importantly, TMP-SMZ resistance was low in the current study. Based on this information, we feel confident recommending TMP-SMZ as the first choice for the treatment of nocardiosis in Iran. Linezolid broadly covers *Nocardia* and would be a second choice, although the costs are considerably greater. We strongly recommend that drug sensitivity testing is helpful in all patients with serious disease.

## Additional Information

**How to cite this article**: Hashemi-Shahraki, A. *et al.* Genetic diversity and antimicrobial susceptibility of Nocardia species among patients with nocardiosis. *Sci. Rep.* **5**, 17862; doi: 10.1038/srep17862 (2015).

## Figures and Tables

**Table 1:** Shows the demographic information of study population.

	Pulmonary nocardiosis N(%)	Extrapulmonary nocardiosis N(%)	P-value
Age (mean ± SD)	55.8 ± 20	38.6 ± 19	<0.0001
Sex			0.760
Female	35(53)	34(56)	
Male	31(47)	27(44)	
Underlying condition			
Healthy	28(42)	28(46)	0.693
HIV*	7(11)	6(10)	0.886
Solid organ transplant	6(9)	5(8)	0.858
Diabetes	3(5)	4(7)	0.620
COPD**	6(9)	5(8)	0.858
Corticosteroid therapy	1(2)	8(13)	0.011
Others	15(22)	5(8)	N/A
Chest radiograph			N/A
Nodular or consolidative opacities	37(56)	N/A	
Cavitary lesion	18(30)	N/A	
Pleural effusion	8(14)	N/A	

HIV: The human immunodeficiency virus, **COPD: chronic obstructive pulmonary disease, N/A: not applicable.

**Table 2:** Nocardia species isolated from human infection in Iran.

	Pulmonary nocardiosis	Extrapulmonary nocardiosis
Most common isolated species	*N. asteroides*	*N. cyriacigeorgica*
Commonly isolated species	*N. farcinica*	*N. abscessus*
	*N. cyriacigeorgica*	*N. farcinica*
	*N.otitidiscaviarum*	*N. asteroides*
Frequently isolated species	*N. abscessus*	*N. wallacei*
		*N.otitidiscaviarum*
Rarely isolated species	*N. nova*	*N. nova*
	*N. wallacei*	*N. carnea*
	*N. arthritidis*	
	*N. carnea*	
	*N. veterana*	
	*N. kruczakiae*	

Commonly isolated was defined as frequency rate >10%, frequently isolated was defined as frequency rate between 10% and 3%., rarely isolated was defined as frequency <3%.

**Table 3:** Drug susceptibility testing results for clinical isolates of *Nocardia*.

Species (number of isolates)/antibiotics	MIC (μg/ml)		Number (%) of isolates		
	50%	90%	Susceptible	Intermediate	Resistant
***N. cyriacigeorgica*****(25)**					
Amikacin	0.125	1	25(100)	—	0 (0)
Amoxicillin-clavulanic acid	8	64	0(0)	5(20)	20(80)
Ceftriaxone	4	64	20(80)	2(8)	3(12)
Ciprofloxacin^a^	4	32	0(0)	7(28)	18(72)
Clarithromycin^b^	2	8	8(32)	7(28)	10(40)
Cefepime	0.125	0.5	25(100)	0(0)	0(0)
Cefotaxime	4	64	20(80)	1(4)	4(16)
Gentamicin	1	2	25(100)	0(0)	0(0)
Doxycycline	0.25	16	18(72)	3(12)	4(16)
Imipenem	1	64	15(60)	2(8)	8(32)
Linezolid^c^	0.125	0.5	25(100)	—	—
Minocycline	8	32	0(0)	3(8)	22(88)
Moxifloxacin	32	64	0(0)	0(0)	25(100)
Tobramycin	0.125	0.5	25(100)	0(0)	0(0)
TMP-SMZ	0.125	0.5	25(100)	—	0(0)
***N. asteroids*****(31)**					
Amikacin	2	16	24(77)	—	7 (23)
Amoxicillin-clavulanic acid	16	32	17(55)	3(10)	11(35)
Ceftriaxone	4	128	17(55)	3(10)	11(35)
Ciprofloxacin^a^	0.125	8	17(55)	5(16)	9(29)
Clarithromycin^b^	16	32	2(6)	3(10)	26(84)
Cefepime	16	64	12(39)	5(16)	14(45)
Cefotaxime	32	64	10(32)	9 (29)	12(39)
Gentamicin	0.125	4	28(90)	0(0)	3(10)
Doxycycline	0.125	32	16(52)	7(23)	8(25)
Imipenem	1	32	28(90)	0(0)	3(10)
Linezolid^c^	0.125	1	31(100)	—	—
Minocycline	8	32	8(25)	10(32)	13(43)
Moxifloxacin	8	16	12 (39)	8(25)	11(35)
Tobramycin	4	16	19(61)	5(16)	7(23)
TMP-SMZ	0.5	1	31(100)	—	0 (0)
***N. farcinica*****(26)**					
Amikacin	0.125	1	26(100)	—	0 (0)
Amoxicillin-clavulanic acid	2	32	16 (61)	2(8)	8(31)
Ceftriaxone	128	256	0 (0)	0(0)	26(100)
Ciprofloxacin^a^	0.125	2	10(38)	5(20)	11(42)
Clarithromycin^b^	8	32	0(0)	4(15)	22(85)
Cefepime	32	64	0(0)	4(15)	22(85)
Cefotaxime	32	64	0(0)	0(0)	26(100)
Gentamicin	32	128	0(0)	0(0)	26(100)
Doxycycline	16	32	0(0)	0(0)	26(100)
Imipenem	1	32	15(58)	3(11)	8 (31)
Linezolid^c^	0.125	1	26(100)	—	—
Minocycline	8	32	0(0)	0(0)	26(100)
Moxifloxacin	4	16	10(38)	3(11)	13(51)
Tobramycin	16	32	0(0)	0(0)	26(100)
TMP-SMZ	0.5	8	24(92)	—	2(8)
***N. otitidiscaviarum*****(12)**					
Amikacin	0.125	2	12(100)	—	0(0)
Amoxicillin-clavulanic acid	32	64	0(0)	0(0)	12(100)
Ceftriaxone	64	256	0(0)	2(17)	10(83)
Ciprofloxacin^a^	4	8	2(16)	3(25)	7(58)
Clarithromycin^b^	4	16	4(33)	1(8)	7(58)
Cefepime	16	32	4(33)	4(33)	4(33)
Cefotaxime	64	128′	2(17)	1(8)	9(75)
Gentamicin	2	32	6(50)	0(0)	6(50)
Doxycycline	8	32	1(8)	1(8)	10(83)
Imipenem	16	64	0(0)	0(0)	12(100)
Linezolid^c^	0.125	1	12(100)	—	—
Minocycline	8	32	1(8)	1(8)	10(83)
Moxifloxacin	4	16	3(25)	3(25)	6(50)
Tobramycin	0.5	1	12(100)	0(0)	0(0)
TMP-SMZ	0.125	0.5	12(100)	—	0(0)
***N. abscessus*****(19)**					
Amikacin	16	32	7(37)	—	12(63)
Amoxicillin-clavulanic acid	4	64	8(42)	2(11)	9(47)
Ceftriaxone	0.5	1	19 (100)	0(0)	0(0)
Ciprofloxacin^a^	4	16	0(0)	0(0)	19(100)
Clarithromycin^b^	4	16	6(31)	3(16)	10(53)
Cefepime	8	32	10(53)	3(16)	6(31)
Cefotaxime	8	64	10 (53)	3(16)	6(31)
Gentamicin	0.5	1	19(100)	0(0)	0(0)
Doxycycline	1	8	4(21)	3(16)	12(63)
Imipenem	8	32	4(21)	2(11)	13(68)
Linezolid^c^	0.125	1	19(100)	—	—
Minocycline	4	16	5(26)	2(11)	12(63)
Moxifloxacin	4	16	0(0)	0(0)	19(100)
Tobramycin	0.5	1	19(100)	0(0)	0(0)
TMP-SMZ	0.125	0.5	19(100)	—	0(0)

^a^Ciprofloxacin may be used as a class representative for the older fluoroquinolones: ciprofloxacin, ofloxacin, and levofloxacin.

^b^Class representative for newer macrolides.

^c^Proposed breakpoint with linezolid MIC values >8 μg/mL for *Nocardia* isolates have been adapted from reference^37^. Breakpoints are arbitrary since there are currently no NCCLS interpretive criteria.
